# Let Them Eat Plants! Two Arguments for Raising Children on a (Predominantly) Plant-Based Diet

**DOI:** 10.1007/s11158-025-09728-9

**Published:** 2025-08-21

**Authors:** Sabine Hohl, Angela K. Martin

**Affiliations:** 1 Universität Basel, Philosophisches Seminar, Steinengraben 5, 4051 Basel, Switzerland; 2 University of Fribourg, Environmental Sciences and Humanities Institute, Ch. du Musée 4, 1700 Fribourg, Switzerland

**Keywords:** Veganism, Plant-based diet, Animal ethics, Environmental ethics, Children, Family ethics, Moral education

## Abstract

In this article, we present two independent arguments in favor of the view that parents have a *pro tanto* moral duty to feed their children a predominantly plant-based diet. The first is the ‘Animal Harm Argument’. The significant suffering caused to animals by harmful animal agriculture is morally wrong, and consequently there is a moral duty to avoid consuming products coming from such circumstances. In a family context, parents have a moral duty to teach their young children the best ‘rules-of-thumb’ to comply with morality’s demands. Therefore, parents should, as a rule, provide their children with a plant-based diet. The second is the ‘Environmental Harm Argument’. Consuming animal products is associated with a large ecological footprint. There is a moral duty to reduce one’s ecological footprint, and significant reductions are not feasible without substantially cutting down on animal products, so moving toward a more plant-based diet is morally required. In families, parents have a special responsibility to help reduce their children’s ecological footprint, and to prepare them to heed laws requiring such reductions in the future, and therefore should feed them a predominantly plant-based diet. Lastly, we address several objections to our arguments and show why they do not hold.

## Introduction

Parents and other caregivers serve children countless meals throughout childhood. But what should these meals contain? In this article, we present two arguments why parents should provide their children with predominantly plant-based meals^1^—for the sake of sentient animals, and for the sake of the environment. This position is not without controversy. Parents who feed their children a plant-based diet often face public criticism. They may be reproached for unjustly ‘imposing’ their own vegan lifestyle on their children, exposing them to social ostracism, or endangering their children’s health.^2^ Indeed, plant-based diets for children are frequently criticized due to health concerns, which have been fueled by rare cases where vegan children did suffer severe harm. Nevertheless, several major Academies of Nutrition, such as those in the United States, Canada, and Australia, have declared that plant-based diets are safe for all stages of life, including childhood, provided that they are well-planned (e.g., with adequate supplementation of vitamin B12).^3^ The World Health Organization (WHO) also recommends a shift toward plant-based diets, while paying due attention to adequate micronutrient intake (WHO Regional Office for Europe 2021). This institutional support bolsters the claim that implementing a well-planned plant-based diet for children is at least morally *permissible*. But could it even be morally *obligatory*?

When it comes to parental responsibilities regarding children's diet, it is natural to consult the literature on family ethics. Some philosophers have argued that parents have a duty to facilitate their children’s moral development as part of their general fiduciary obligations (prominently defended by Brighouse and Swift (2014); see also Clayton (2006) and Fowler (2020) on parental obligations concerning children’s moral development in general). Below, we present three arguments based on children's moral development and their role as future moral agents that have implications for how parents should feed their children.

Elizabeth Cripps (2017) argues that parents have a responsibility to raise what she calls “good global climate citizens” (Cripps 2017, p. 41).They must prepare their children to fulfill climate justice duties as adults. Cripps’s argument is based on the importance of valuing the child as a member of the same moral community as oneself (2017, p. 48 ff.). While everyone should value children in this way, parents have a special relationship with their children that gives them the opportunity and the obligation to facilitate and nurture their abilities to become good global climate citizens. A parent who does not foster the moral development of their child to enable them to respond appropriately to climate change would be failing a parental duty. One such appropriate response to climate change may be to move to a predominantly plant-based diet.^4^

Also taking children’s status as future moral agents as his starting point, Daniel Butt makes a more specific argument in favor of feeding children a vegetarian diet. Butt argues that “[…] parents have a morally significant reason not to feed meat to their children, which stems from their fiduciary responsibility for the child’s moral development” (Butt 2021, p. 981).^5^ Importantly, Butt does not assume that eating meat is morally wrong. However, he asks, what if children, once they have become teenagers or adults, turn to ethical vegetarianism? They may feel that having been fed meat as children had a corrupting effect on their moral integrity. For example, once they start developing moral responsibility, they may not be able to immediately adopt a vegetarian or vegan diet because they are so used to animal products. Their parents’ actions have made them more likely to fail at adopting a vegetarian diet—a moral failure, in their eyes. Parents, so the argument goes, should avoid this risk by abstaining from feeding children meat in the first place.

In a similar vein, and arguing most specifically for a “maximally plant based diet” for children, Jeremy Fischer and Rachel Fredericks have recently argued that children’s interests give rise to a duty to raise them as plant-based as possible (Fischer and Fredericks 2024, p. 2). These interests concern children’s moral development, autonomy, and physical health.^6^ In particular, Fischer and Fredericks argue that “carnist” beliefs correlate with a rejection of human out-groups and affirmation of social hierarchies.^7^ Furthermore, the cognitive dissonance and motivated reasoning often underlying carnism are harmful to children’s intellects, as are attempts to mislead children about where animal products come from.

These recent contributions to the debate have in common that they focus on parental duties that are based on *children’s* interests in moral development, rather than any *third parties’* interests—although, to be sure, the development of children’s moral character and agency is also of high relevance to the moral community. In contrast, this article explores the following question: What parental duties regarding children's diets arise from considerations of *harm to others*? One may wonder why such an argument has not made an appearance in the family ethics literature so far—after all, harm to others looks like one of the most straightforward reasons for a plant-based diet. The reason for this, we believe, is that in the family ethics literature, diet has been considered a typical ‘lifestyle’ issue about which there can be reasonable disagreement within a liberal framework. Not eating animal-based food may be part of some conceptions of the good, but equally reasonable conceptions of the good may endorse eating meat and other animal products. It is not obvious that parents have the moral permission to teach their children any conception of the good they happen to endorse. Therefore, if a plant-based diet is part of such a conception of the good, rather than a matter of justice, it is not clear that parents may teach it to their children and feed them accordingly. For example, Matthew Clayton (2006) argues that parents must stay neutral between different conceptions of the good so as not to violate their children’s independence. We think that it is a mistake to categorize diet as a lifestyle question, however. There is good reason to consider it a matter of justice instead.

To establish this, we focus on two points: i) the significant and unnecessary suffering involved in animal agriculture as it is currently practiced (the Animal Harm Argument); and ii) the link between plant-based diets and lower greenhouse-gas emissions (the Environmental Harm Argument). To a certain extent, these arguments have been discussed in the animal ethics and environmental ethics literature before. For example, Carlo Alvaro (2019) argues that the moral reasons for raising children on a vegan diet outweigh the reasons for omnivorous diets. In his response to an article by Marcus Hunt (2019), he shows that well-planned plant-based diets may be healthier than omnivorous diets and contends that vegan children are not more ostracized than other children. In addition, he posits that vegan diets cause less harm to animals and to the environment. However, Alvaro does not explore the specific *parental duties* that might result from this. Now, one might think that if these harm-based arguments are sound, then it follows that they are also sound for children, and that parents must therefore feed their children a plant-based diet. We do not disagree, but in our view, there is more to the argument. Parents in the Global North, we contend, have *additional* harm-based reasons to implement a plant-based diet for their children—reasons that actually go above and beyond the reasons that apply to their own consumption as adults. In particular, parents in the Global North ought to teach children useful rules-of-thumb (Animal Harm Argument) as well as prepare their children for a low-emission world (Environmental Harm Argument).^8^

We proceed as follows: we first present the ‘Animal Harm Argument’ in favor of feeding children a plant-based diet, followed by the ‘Environmental Harm Argument’, and finally we address several objections to our proposal.

## The Animal Harm Argument

There is widespread disagreement about the ethics of eating animal products.^9^ While many animal ethicists have questioned and challenged the practice of eating meat and other animal-derived products from an ethical perspective,^10^ the general public seems reluctant to reconsider their consumption of meat, dairy, etc.—in fact, the consumption of animal products globally is rising.^11^ Correspondingly, we start from the assumption that there exists a pluralism of views in society regarding the morality of eating animal products.

### The Harm Involved in Animal Agriculture and its Alternative

Animal agriculture, as it is currently practiced in most parts of the Western world, undeniably causes significant suffering to animals. Animals destined for the food industry, such as pigs, chickens, and cows, are often kept *en masse* in dreadful living conditions. Moreover, they are bred with traits which increase their yield, but which decrease their welfare. For example, dairy cows bred to maximize milk production frequently suffer from udder infections (Cheng and Han 2020); pigs, turkeys, and other animals are bred to gain as much weight as possible in the shortest time, which puts immense strain on their legs and disrupts their reproductive functioning (see, for example, Prunier et al. 2010; Hoel et al. 2016). Moreover, most farming practices exact a heavy psychological toll: the boredom and restlessness the animals experience in densely populated barns results in aggression, even cannibalism (Meagher 2019; Estevez et al. 2007; Rodenburg and Koene 2007). Dairy cows are often separated from their offspring in the first few weeks of life, causing emotional distress (Neave et al. 2024; Stěhulová et al. 2008). When farmed animals reach their slaughter age—often a decade or more before their lifespan would naturally end—they undergo long, stressful transportation to the slaughterhouse. As slaughterhouses attempt to kill as many animals as possible, as quickly as possible, it sometimes happens that the killing method fails (Wenzlawowicz et al. 2012) and animals are skinned alive, enduring an excruciating death.

It might theoretically be possible to breed, raise, and kill animals for food without inflicting harm. Imagine, for example, that animals are kept under free-range conditions, where they can roam freely, form social bonds, and pursue species-typical behavior. Farmers have enough time to look after individual animals, fostering their welfare. When the animals are older, they are killed directly on the farm, avoiding any transport-related stress. While many animal ethicists would contend that even these ideally bred and reared animals are still wronged insofar as they are killed prematurely (Cochrane 2012; Cooke 2023; Singer 2011; Regan 2004), a majority of consumers likely would prefer animal products from such farms—despite the likely higher prices—as such animal-welfare oriented farming practices would probably be deemed more ethically acceptable.^12^ Thus, put in ethical terms, most people recognize that it is morally wrong to harm sentient animals unnecessarily, and would agree that there is a *pro tanto* duty to avoid causing harm to animals when it can be avoided or at least be minimized. We thus assume here that there is agreement about the wrongfulness of *unnecessarily* causing significant animal suffering.

Most current farming practices are far removed from the ideal sketched above, however. And even if non-harmful practices for breeding, rearing and killing animals are possible (for example, on small-scale family-farms), the reality is that the overwhelming majority of people do not produce their own food from locally raised animals who lead a good life and who are eventually slaughtered on the farm they live on, nor do most consumers buy such products. Instead, the vast majority of people rely on supermarkets, markets, and restaurants. As for animal welfare, they can only trust the information provided by the food industry; as a result, it is difficult for consumers to determine whether the animal products they buy were harmfully produced or not—especially in a globalized world with food on the go and multiple production chains that are not easily traceable. At the end of the day, most consumers cannot really discern whether they are buying their animal products from an ethically problematic source or not.

There is, of course, a clear alternative to harmful agriculture: consumers could abstain from buying and eating animal products altogether. Consuming animal products is not an alternative-free scenario, especially not in the Global North where an abundance of healthy and varied plant-based products and supplements are readily available. The considerable harm that most farming practices cause to animals can be avoided if people adopt a plant-based diet.^13^

Consequently, given the availability of healthy plant-based alternatives, consumers who buy harmfully produced animal products are, in our view, morally *complicit* in the wrongs caused by harmful animal agriculture. By buying harmfully produced animal products, consumers actively contribute to the moral wrongs of harmful animal agriculture—perpetuating them through further demand for products involving considerable animal suffering. To avoid this moral complicity, consumers must face their moral obligation to refrain from buying and consuming these products in the first place, whenever possible.

Many consumers may doubt though that they are morally complicit in any of the moral wrongs occurring in the production of goods they buy, based on the following reasoning: whether *they* buy a given product or not is not going to make any difference to the outcome, their purchases being no more than a drop in the ocean (for a forceful expression of this view, see Sinnott-Armstrong 2005). It can be hard to see how there can be moral responsibility for an outcome that one cannot individually prevent. This is known as the “problem of collective harm” or the “moral inefficacy objection”.^14^

However, there are several possible bases for indeed considering consumers to be morally responsible for the harms that occur in the production of the goods they buy. Candidates include the presence of a positive (though often tiny) probability of changing outcomes as an individual consumer (Kagan 2011; Isaacs et al. 2024), or causal contributions that do not necessarily entail a positive probability of changing outcomes (Braham and van Hees 2012; Hohl 2017). From a deontological perspective, in turn, consuming more than one’s fair share of environmental goods can be considered morally problematic (Baatz 2014). And a further basis for moral responsibility may be found in intentional participation in a collective wrong (Kutz 2010).

For present purposes, we do not take a position on which account of individual moral responsibility for collectively caused harms is the right one, but we assume for the sake of the argument that there is a way to overcome this issue and to identify people as morally complicit in collectively caused harms based on their consumption choices.

### The Parental Duty to Feed Children a Plant-Based Diet

Parents are supposed to teach their children the basics of morality throughout childhood, helping them grow into decent, respectful adult citizens. Moral lessons are often taught in the form of rules-of-thumb that comply with morality’s demands (Mammen and Paulus 2023). For example, although there may be situations in which it might be permissible—or even morally required—to lie, the rule-of-thumb for most situations is that one should refrain from lying. Another example is hitting other people. Generally, morality demands that we not hit other people; while there may be exceptions to this rule (such as self-defense), for the most part, it is sensible to teach children the rule of not hitting each other. A third example is sharing: as a rule-of-thumb, we usually teach our children to share with others (e.g., their toys, snacks, etc.).^15^ These rules-of-thumb guide young children as they develop their moral character and acquire sound moral habits. As children mature, these rules-of-thumb can be refined with further details and nuanced with exceptions. For instance, parents can explain to their children that we should not lie, but that there are exceptions to this rule (e.g., lying to someone to prevent them from harming others), leading to a more refined rule about the circumstances in which it is appropriate to lie.

Besides sharing, not lying, and not hitting others, a further demand of morality is to abstain from causing any unnecessary and unjustified suffering to animals. Most parents automatically teach this rule to their young children, especially in the case of companion animals. For example, parents tell their children not to pull the family dog’s tail or not to hit neighborhood cats. There may be various reasons for reprimanding children for such behaviors (such as avoiding situations where an aggravated dog or cat might bite the child), but the fundamental reason why parents aim to teach such a rule is that it is bad in and of itself to harm an animal unnecessarily. This is also shown by the fact that most parents are committed to teaching their children the rule-of-thumb that it is morally impermissible to harm animals who are not considered companion animals. For example, many parents teach their children that it is impermissible to throw rocks at grazing cows or sheep or to set pigeons on fire. That is, most parents are committed to teaching their children the moral rule that it is *generally* morally impermissible to harm animals unnecessarily—regardless of the species of the animals.

By this reasoning, and for the sake of consistency in children’s moral education, the same rule-of-thumb should be extended to the animals used for animal products. If it is a moral imperative to teach young children that unnecessarily harming animals is wrong, and if current agricultural practices unnecessarily harm animals, then parents should teach their children that morality requires them to abstain from consuming harmfully produced animal products. To comply with the moral duty to avoid causing unnecessary harm to animals, parents should act as follows: explain to their children that, as a rule-of-thumb, they should eat plant-based foods; feed their children a plant-based diet; and eat a plant-based diet in front of their children.

The Animal Harm Argument can be summarized as follows:

1. Animal agriculture that causes significant and unnecessary harm to animals is morally wrong.
2. By buying products associated with significant and unnecessary harm to animals, consumers actively contribute to these harms and become morally complicit in them.
3. There is a moral *pro tanto* duty for consumers to avoid animal products that involve unnecessary and significant harms to animals.
4. A large majority of currently available animal products come from circumstances that cause significant and unnecessary harm to animals; these products are indistinguishable from non-harmful animal products.
5. Parents have a duty to teach their young children the best ‘rules-of-thumb’ to comply with morality’s demands.
6. In practice, teaching young children the rule-of-thumb to adopt a plant-based diet is required to fulfill the *pro tanto* duty to avoid animal products associated with           significant and unnecessary harm to animals.
7. Therefore, parents should teach their children the rule-of-thumb to adopt a plant-based diet.

     

We have argued for P1, P2, P3, and P4 above. Let us say more about P5. An adult may be able to distinguish between meat from ethical sources (e.g., from free-roaming cattle who were killed on a family-run farm, from dumpster-diving, or from roadkill) and non-ethical sources (e.g., factory-farmed meat). However, most young children are likely incapable of making this distinction, as the product will look and taste the same to them. Teaching children to eat plant-based food by default helps them build a morally important habit, without suggesting to them that it is acceptable to eat products which come from harmful farming practices. Many parents who, in principle, care about the source of their animal products will likely still buy factory-farmed animal products in restaurants and on the go, where the sources cannot be traced. But through practices such as these, they teach their children that the source of animal products does not really matter, which seems to imply that it is morally permissible to harm animals. Adopting a plant-based diet *in front of children* gets rid of this problem and teaches them that harming animals is *pro tanto* morally wrong.

Teaching young children the rule-of-thumb to eat plant-based food offers several advantages. It provides them with an easy-to-follow rule that aligns with the moral obligation to avoid harming animals. If parents articulate this moral duty to children and explain why it results in the rule-of-thumb to eat plant-based food, children grasp a straightforward rule about the morality of eating meat products of unknown origin. Consequently, children will be more likely to understand that animals often endure significant suffering in the meat industry, and that this suffering constitutes a moral wrong to be avoided. Furthermore, if children decide to become ethically motivated vegans later on in life, their transition costs will be negligible: they will not face any major difficulties in adopting a fully vegan lifestyle, as they will already have formed the habit of avoiding a large class of animal-based products. Moreover, once they grow up, children will not have to resolve the meat paradox—a cognitive dissonance caused by the tension between one’s wish not to cause any suffering to animals and one’s desire to eat animal products.^16^ A further advantage of our proposal is that children will not retroactively feel complicit in the moral wrong of having caused unnecessary suffering to animals once they turn into adults, as they will have eaten plant-based during childhood (Butt 2021).

One may object that our argument applies only to a limited age group. When children are very young, they cannot understand moral rules-of-thumb, much less their justification, and so teaching them to eat a plant-based diet as a rule-of-thumb does not make sense. Our argument implies that adopting a plant-based diet only makes sense as a moral rule-of-thumb if the child is capable of broadly understanding general moral rules. If this only becomes possible starting at approximately one-and-a-half or two years old, then before that age, it may be ethically justified to feed meat to very young children, as long as they do not develop a habit and then actively demand meat later on, when they *are* capable of understanding moral rules.

Conversely, when children are older (say, age eight and older), they are likely capable of understanding exceptions to a rule. That is, children are capable of grasping that the specific source of animal-derived products matters from a moral perspective. This means that when the origin of animal products is clearly known (e.g., the animal comes from a neighboring farm where one knows that the animals are treated well), it may be justified to offer these animal products to older children, along with an explanation of why *this* meat is ethically permissible. This also means that other methods to teach morality, such as Socratic, debate-based approaches, may become more appropriate. In other words, our argument does not rule out the possibility that parents have a duty to help older children actively develop the skills needed to distinguish between different sources of animal products.

This narrows the age range for the rule-of-thumb-based Animal Harm Argument to children from toddlerhood up to approximately around seven or eight years old. However, our argument nonetheless has merit. Its endorsement would avoid the habituation of young children to animal-based products and thus the development of particular gustatory preferences. In turn, this would facilitate debates about animal-based products with older children without specific preconceptions.^17^

## The Environmental Harm Argument

Our second argument goes as follows: raising children on a predominantly plant-based diet is necessary to significantly reduce children’s environmental footprint; and significantly reducing one’s environmental footprint is morally required for those living in affluent countries with unsustainably high environmental footprints. First, we put forward an argument concerning children’s environmental footprints during childhood: given that children are not yet moral agents, it is their parents who are morally responsible for significantly reducing their children’s environmental footprints. Second, we present two arguments concerning children’s environmental footprints as future adults, contending that parents have a moral duty to raise their children in a way that enables them to have a (more) sustainable footprint as future adults. Raising children on a plant-based diet is not only one of the most effective ways to accomplish this aim, but is also integral to it.^18^ From a moral point of view, therefore, parents ought to maintain a (predominantly) plant-based diet for their children.

### Environmental Footprints and their Moral Relevance

Environmental footprints measure an individual’s impact on the environment. Different versions of environmental footprints include different elements (such as greenhouse gas emissions, land use, water use) and measuring methods; no environmental footprint will *exactly* predict the actual impact of any individual’s behavior. Rather, environmental footprints represent the *average* impact of certain kinds of behavior, such as eating a specific kind of diet, using certain means of transportation, and the like.

An individual’s environmental footprint is morally relevant: if one lives a lifestyle that, if followed by most people, would lead to significant environmental harm, then one is overusing natural resources. From a moral perspective, such overuse is unfair: nobody has a right to more than their fair share of natural resources.^19^ By overusing, one either forces others to use less than their fair share, or one accepts the environmental harm caused by collective overuse as a consequence. Both options are morally wrong: the overuse of natural resources is unjust to those deprived of their fair share; and environmental harm caused by overuse (e.g., the harm caused by anthropogenic climate change) represents preventable and hence unjustifiable harm to others (such as forced migration or death by natural disasters induced by climate change).

One may doubt whether individuals can truly be held morally responsible for their environmental footprints, let alone the collectively caused consequences of unsustainably high environmental footprints (Sinnott-Armstrong 2005). After all, only a portion of one’s footprint can be influenced at the individual level: the rest is determined by the society one lives in and its collective infrastructure. Moreover, one could object that holding individuals responsible for the collectively caused harm of environmental damage and climate change is a problematic shift of collective responsibility onto individuals: individuals are not mainly responsible when it comes to causing and addressing a global problem like climate change, for instance; only collective (political) action has the potential to effectively address large-scale environmental harm. Within the larger picture, individuals’ contributions are mere drops in the ocean.

Nonetheless, these objections do not establish that individual environmental footprints can be discounted *entirely* from a moral perspective. While collective action works best, this does not imply that individuals are completely powerless (Schwenkenbecher 2014; Hohl 2017).^20^ Individuals adopting more environmentally friendly behaviors can be important catalysts for change at the collective level, insofar as individuals can serve as examples for more environmentally friendly lifestyles. Through social contagion, they can influence others and thereby generate significant effects overall. Moreover, successful collective action depends upon individuals’ *willingness* to live with a smaller footprint—something that is often lacking nowadays (as evidenced by the resistance to higher prices for gas, for example). In our view, the dependence of successful collective action on individual willingness to adapt one’s footprint is an underappreciated point. It seems highly unlikely that we will move from a society in which there is no focus at all on individual footprints to one in which collective regulations are imposed upon individuals to significantly reduce individual footprints, thereby forcing lifestyle adaptation. As long as addressing individual footprints is considered ‘taboo’, political actions that impact the former are difficult to implement. Therefore, while individuals’ environmental footprints may not be the most important factor in addressing environmental harm, they should not be underestimated.

It is thus plausible that *some* reduction of affluent Westerners’ individual environmental footprints is morally required. But how much of a reduction? A reasonable target is the amount of carbon emissions per person each year that would maintain the global average of climate-change induced temperature increase at 1.5 degrees, as pledged in the 2015 Paris agreement. An analysis by Oxfam and IEEP estimates the per capita amount compatible with reaching the 1.5 degree goal in the year 2030 at 2.3 tons; the world’s richest 10% are currently on track to emit nearly *ten times* that amount (Oxfam International and the Institute for European Environmental Policy 2021). Consequently, the citizens of affluent countries must make significant reductions to even approach a sustainable per capita footprint.

### The Smaller Environmental Footprint of Plant-Based Diets

The facts are clear: eating a plant-based diet will make one’s individual environmental footprint significantly smaller. Global food production accounts for around 30% of all GHG emissions (Garnett 2011), and a plant-based diet produces the least GHG emissions of all diets. A systematic review of 14 different studies found that plant-based diets reduce diet-related GHG emissions by up to 50% compared to standard omnivore diets (Hallström et al. 2015; see also Burke et al. 2023 for similar results). Moreover, plant-based diets also do well with regard to other aspects, such as land use: “Substituting all animal products with plant-based food can […] reduce the land demand from the diet by up to 60%” (Hallström et al. 2015, p. 6). Another study considered different environmental aspects to build an overall environmental score for various diets, finding a reduction of 69% of this score among people who had adopted plant-based diets compared to those on omnivore diets, with a reduction of 61% for vegetarians (Rabès et al. 2020). Finally, a recent study including over 55,000 individuals in the UK concluded that “the relationship between environmental impact and animal-based food consumption is clear and should prompt the reduction of the latter” (Scarborough et al. 2023, p. 565).

Admittedly, adopting a predominantly plant-based diet is just one of many measures that can reduce one’s environmental footprint; others include refraining from flying, living in a smaller apartment, or not owning a car. That said, it is close to impossible to attain a sustainable environmental footprint as an individual without at least significantly reducing one’s intake of animal products, because animal agriculture accounts for an immense share of overall GHG emissions. In a review of 23 studies of the average GHG emissions associated with different diets, Burke et al. find a daily emission for standard omnivorous diets of 4.83 kg CO_2_ equivalents, which already adds up to over 1.7 tons per year, just for food alone (Burke et al. 2023). In principle, the moral imperative to reduce one’s footprint leaves the means open, but in practice, an effective combination of steps will necessarily include dietary changes toward a plant-based diet. While this state of affairs does not imply a moral duty to adopt a 100% plant-based diet, it does suggest that adopting a predominantly plant-based diet is one of the most effective measures one can take to reduce one’s environmental footprint (in addition to other easily implementable measures, such as not taking planes).

The ‘General Version of the Environmental Harm Argument’ can be summarized as follows:

1. Citizens of affluent countries are morally obligated to significantly reduce their individual environmental footprints.
2. A significant reduction of one’s environmental footprint is not feasible without adopting a predominantly plant-based diet.
3. Citizens of affluent countries are morally obligated to adopt a predominantly plant-based diet.

   

This duty incumbent upon adult citizens of course also extends to parents, which brings us to their specific parental duties with regards to their children’s diet.

### Parents’ Duties to Reduce Children’s Environmental Footprint

Children are not yet morally responsible for their actions. Nevertheless, they consume significant amounts of natural resources and leave an environmental footprint, just as adults do. In our view, parents are morally responsible for the individually influenceable part of their children’s environmental footprints during their childhood. Parents make many decisions that impact their children’s footprint, for example whether they use a car, live in a large house or a smaller apartment, or whether they fly on holiday. And parents are the ones who have the authority to decide what their children eat (at least in the sense of what foods they offer them). Accordingly, adopting a plant-based diet for their children will be one of the most effective ways to significantly lower the latter’s footprint.

These considerations lead us to the ‘Reduction of Children’s Environmental Footprint Argument’:

 P3: Parents are responsible for (the individually influenceable part of) their children’s environmental footprints during their childhood.
C2: Parents in affluent Western countries should make significant reductions to their children’s environmental footprint during childhood.
P2 from above: A significant reduction of one’s environmental footprint is not feasible without adopting a predominantly plant-based diet.
C3: Parents in affluent Western countries are morally obligated to feed a predominantly plant-based diet to their children.

  

Our argument has further implications. Parents also have a duty to ensure that their children, once they reach adulthood, will be well positioned to maintain or at least approach a sustainable environmental footprint. There are two reasons in favor of this view: first, once adults, children will themselves have a moral duty not to exceed their fair share of natural resources. Parents should begin preparing their children for these future duties by initiating them into a low-emission lifestyle as children. By contrast, if children become accustomed to a lifestyle associated with a large environmental footprint, they will have a harder time fulfilling their moral duty as adults, as this will require a difficult lifestyle adjustment. Second, if collective action on climate change is to be successful, new laws must be adopted compelling people to reduce their individual environmental footprints or making it increasingly unaffordable to have a large environmental footprint through taxes (e.g., taxes on meat products). While the adoption of such laws is currently encountering political resistance, as people are accustomed to a large-footprint lifestyle, parents can nonetheless make a significant and indeed necessary contribution toward successful collective action against climate change in the future, by preparing their children for a lower-emission lifestyle. Moreover, equipping their children for life in a new era of collective regulation of GHG-intensive activities is not only morally, but also prudentially sound, as it is reasonably likely scenario that the children of today will *have to* live on a smaller environmental footprint as adults.

This reasoning supplies two further arguments which bolster C3:

‘Preparing Children for their Moral Duties as Adults Argument’:

P4: Parents are morally required to prepare their children for fulfilling their duties regarding their individual environmental footprints once they become adults.
P5: Preparing children to fulfill their duties regarding their individual environmental footprints as adults requires the adoption of a predominantly plant-based diet during childhood.
C3: Parents are morally obligated to provide their children with a predominantly plant-based diet.

   

‘Preparing Children for the Introduction of New Laws Argument’:

P6: Parents are morally required to prepare their children to comply with justified and necessary new laws that are reasonably likely to be imposed in the future.
P7: The future imposition of (justified and necessary) laws requiring a significantly lower environmental footprint is reasonably likely.
P2 from above: A significant reduction of one’s environmental footprint is not feasible without adopting a mostly plant-based diet.
C3: Parents are morally obligated to provide their children with a predominantly plant-based diet.

   

In conclusion, while the moral duty to significantly reduce one’s environmental footprint leaves open how exactly one may achieve this goal, in practice it turns out that adopting a plant-based diet is one of the most effective changes one can make as an individual. Without a significant reduction of one’s consumption of animal products, it will be impossible to even approach a sustainable footprint. Therefore, parents should move toward a plant-based diet for their children. Nonetheless, diets with just small amounts of animal products (e.g., from intensive poultry production) would also be compatible with significantly reducing one’s environmental footprint. Importantly, though, some of the ‘best’ animal products in terms of carbon emissions are especially bad when it comes to animal welfare and would be prohibited by the Animal Harm Argument. That is, if someone is convinced by *both* arguments we have presented here, they have strong reasons to adopt a nearly 100% plant-based diet for their children.

Thus, the Environmental Harm Argument signals a direction—consume (far) fewer animal products—rather than demanding an absolute prohibition of consuming such products. However, we find it important to highlight the Environmental Harm Argument, since many people may still underestimate the impact of their diet on their environmental footprint, and because our argument also stresses the moral value of reductions in animal product consumption that fall short of a 100% plant-based diet.

## Further Clarifications and Objections

In this section, we further clarify our two arguments and address several objections that are likely to be raised against our proposal.

First, one could object that our proposal is too difficult for parents to implement. After all, consistently offering a balanced plant-based diet requires a significant amount of knowledge, planning, effort, and expense. This objection comes in two forms. The first version of this objection focuses on children’s health: in reality, the objection goes, a plant-based diet will *seldom* be well planned enough to be nutritionally balanced (Hunt 2019), as children’s health depends on an adequate intake of certain micronutrients (especially vitamin B12) which are often lacking in plant-based foods. In response, we grant that some parents may fall short of the ideal of a well-planned plant-based diet. We agree that if a child’s parent or caretaker is unable to adhere to pediatricians’ and nutritionists’ recommendations for healthy plant-based diets, then they should not implement a fully plant-based diet for their children. But for most people, meeting the guidelines should be no more challenging than other aspects of parenting. For example, in many countries, parents are already expected to give their small children vitamin D supplements. Thus, it does not seem onerous to add a B12 supplement to this recommendation. Furthermore, in order to be fair, the comparison needs to be drawn between a less-than-ideal plant-based diet and a less-than-ideal standard omnivorous diet (Alvaro 2019), as the latter’s health risks are typically neglected. For example, regular consumption of meat appears to be associated with an increased overall cancer risk (WHO Regional Office for Europe 2021; Segovia-Siapco and Sabaté 2019). Nonetheless, children’s regular consumption of meat as part of a less-than-ideal omnivore diet is usually not discussed as problematic—an omission that contrasts with the scrutiny to which plant-based diets are subjected. Finally, a plant-based diet also has health *advantages* that children who eat meat and dairy are missing out on, chief among them a lower obesity risk (WHO Regional Office for Europe 2021). There is not much reason to think that an average child raised on a plant-based diet will be less healthy than an average child raised on an omnivorous diet. The greatest danger of a less-than-ideal plant-based diet is probably a deficiency of vitamin B12, which is a problem for other groups as well, not just vegans (although particularly pronounced in the latter). Adding B12 to plant-based products, such as soy milk or cereal, would help address this problem effectively.

The second version of the objection focuses on the supposed overdemandingness of plant-based diets: in an omnivorous world, implementing a plant-based diet will cause parents hardship, financial or otherwise, thereby representing an excessively onerous moral demand. In response to the financial worries in particular, we point out that a plant-based diet is likely no more expensive than an omnivorous diet, in most places. Lentils, beans, and other staples for plant-based diets are cheap, whereas expensive meat-replacement products are typically unnecessary. Depending on where one lives, the availability of plant-based products for takeout, at restaurants etc. may of course be better or worse (for instance, it is certainly easier to adopt a plant-based diet in big cities than in the countryside). Nonetheless, for many parents, implementing a predominantly plant-based diet at home is doable and is getting easier each passing year.^21^ Schools and daycare currently pose a bigger issue, as not all of them offer plant-based meals yet. But parents could, in this case, at least try to raise the issue and lobby for changes at those institutions together with like-minded people who recognize the kinds of environmental and animal welfare concerns we have articulated above.

A second objection raises the possibility of children being harmed by the social ostracism they might experience by “outing” themselves as vegan (Hunt 2019). Anecdotally, many parents (even vegetarians and vegans) do seem to default to an omnivorous diet for their children in social situations and at school or daycare, out of fear that they might otherwise miss out on something or experience negative social interactions. Such behavior suggests a worry about harming their children’s social lives by imposing a currently still atypical plant-based diet on them.

To this worry, we have a practical response and a principled one. The practical response is as follows: the feared negative effect may not occur at all, whereas overcoming this social worry may help with avoiding vicious cycles that prevent progress. If every parent who would like to implement a more plant-based diet for their child is held back by fear of social ostracism, social progress will be all the more difficult to achieve. At the very least, parents should try it out first and see what actually happens, rather than assuming a negative outcome beforehand. The more principled response is as follows: sometimes doing the right thing comes at a cost. We do not think that parents must entirely shield their children from social stresses. The situation with plant-based diets bears some similarities to, e.g., queer parents deciding whether they want to be open about their status at school or in their child’s social circle. Admittedly, such a decision *could* lead to negative experiences for their child. On the other hand, the courage of forerunners and increasing social acceptance have made the situation less difficult over time. By this we do not mean that parents *must* encourage their children to present themselves with the label “vegan” or “plant-based”, only that it certainly seems morally permissible for them to do so, even if a child may experience some social blowback. Just as parents are not obligated to have children hide their religious status or their membership in a rainbow family in order to protect them, neither are they obligated to make their children hide their plant-based diet.

A third objection runs as follows. Even if one accepts our argument that *children* should be fed predominantly a plant-based diet, this does not imply that *parents* must similarly restrict themselves. While this objection is broadly correct, it requires clarification. Parents serve as role models, so only feeding their children plant-based meals while themselves openly eating animal-derived products in front of the children would likely lead to confusion. Therefore, parents should maintain a plant-based diet *in front of* their children. However, this does not imply that parents must adhere to a strictly plant-based diet when *away* from their children. In theory, it may be ethically justified for the parents to eat animal products during their lunch break at their work cafeteria or when they are on vacation without their children. In essence, parents who are aware of the source of animal products might justifiably consume them, but not in front of their children. Note, though, that the same presumptions about the ethics of consuming such products also hold for adults.

## Conclusion

In this article, we have presented two independent arguments in favor of parents’ moral duty to offer their children a (predominantly) plant-based diet. First, there is a moral duty to avoid contributing to—and *ipso facto* being morally complicit in—the enormous, unnecessary suffering many animals endure due to current farming practices. Parents ought to shoulder this duty on their children’s behalf during their childhood, and they ought also to promote plant-based eating as part of their more general role of teaching their children rules of morality and preparing them to eventually come of age into moral agency. Even if there exist some animal products which do not involve significant animal suffering, to children’s eyes they remain indistinguishable from products which do involve animal suffering. Accordingly, we have argued that adopting a plant-based diet is the best rule-of-thumb to teach to children. Second, adopting a mostly plant-based diet is also an effective means of significantly reducing one’s individual environmental footprint. Parents are morally responsible for their children’s individually influenceable footprints during childhood and for preparing them for a low-emission lifestyle once they become adults. Parents’ fulfilling these responsibilities, we have contended, necessarily involves implementing a mostly plant-based diet for their children.

## Footnotes

1 For simplicity, we use the terms ‘plant-based’ and ‘vegan’ interchangeably. Some may claim that children cannot properly be ‘vegans’ insofar as veganism is conceived as a form of political boycott against systematic animal exploitation (Cochrane and Cojocaru (2023), whereas young children are incapable of being politically motivated agents. For our purposes, it suffices to understand being vegan as “the abstention from the consumption of animal-derived products” (Dutkiewicz and Dickstein (2021, p. 1).

2 E.g., Hunt (2019). For a response to these criticisms, see Alvaro (2019). Note that this discussion between Hunt (2019) and Alvaro (2019) led to several articles with responses, such as Hunt (2021); Milburn (2022); Alvaro (2020).

3 Melina et al. (2016); National Health and Medical Research Council (2013); Position of the American Dietetic Association and Dietitians of Canada (2003). In addition, see Craig et al. (2021).

4 Cripps (2023, p. 144) indeed endorses the view that parents should move toward a more plant-based diet for climate-related reasons, but she does not defend a *moral duty* to raise children on a plant-based diet.

5 Butt (2021) mentions at the end of his article that his argument could be extended to animal products other than meat.

6 According to Fischer and Fredericks (2024), plant-based diets may reduce certain risks to one’s physical health (such as overall risk of cancer or type 2 diabetes) posed by diets that include regular consumption of meat.

7 ‘Carnism’ is a belief system or ideology that conditions people to eat certain animals (Joy 2011).

8 An anonymous reviewer wondered whether such duties may also extend to caregivers other than parents. While we consider this plausible in many cases, it is less clear whether non-parental caregivers possess the required authority to make decisions on behalf of a child. Therefore, we concentrate on parental duties.

9 It is not possible to list the whole literature dealing with the ethics of eating animals; for an overview of some of the debates, see, for example, Bramble and Fischer (2016); Abbate (2024); Doggett (2018); Fischer (2020); Milburn (2023).

10 To name a few, see, for example, Regan (2004); Singer (2009, 2011); Cochrane (2012); Francione (2021); Bruers (2015); McPherson (2014, 2018).

11 See Ritchie et al. (2023); United Nations Food and Agriculture Organization (2023).

12 See, for example, Giannetto et al. (2023); Yang (2023); Heng et al. (2013); European Consumer Organisation (2024); Alonso et al. (2020); Fonseca and Sanchez-Sabate (2022); Richter et al. (2025). However, price-sensitive consumers are not so ready to pay higher prices for animal products from better farming practices; see, for example, Gorton et al. (2023); Hyland et al. (2022). It has to be noted, though, that many consumers claim that they want to buy animal-friendly products, but fail to do so in practice. We thank an anonymous reviewer for highlighting this issue. We cannot explain this contradiction away. Psychological studies are needed that investigate if this contradiction is due to unclear labeling, ignorance, akrasia, or the like.

13 One may object that humanely reared animals benefit from being alive, and not eating them thus deprives them of the benefits of their being brought into existence in the first place (for a discussion of this argument, see McMahan 2008). However, for our argument’s sake, the *uncertainty* as to whether the animal was humanely raised or not matters. Given that people are often unsure whether the animals they eat had a life worth living or not, it is better to err on the safe side and avoid feeding animal-derived products to children.

14 For further discussion of this problem also see, for example, McMullen and Halteman (2019); Budolfson (2019); Fischer (2020).

15 The psychological literature on children’s early moral development has established that young children (from around age three) indeed follow social and moral norms and even help enforce them when they are violated (see Vaish and Tomasello (2014), Smith et al. (2013). On the important role of interactions in the family for children’s moral development, see Dunn (2014).

16 For a discussion of the meat paradox from philosophical and psychological points of view, see, for example, Ursin (2016); Jaquet (2021); Aaltola (2019); Dhont and Hodson (2019); Rothgerber and Rosenfeld (2021). Some studies show that children are less speciesist than adults and sometimes prioritize animals over humans (Wilks et al. (2021); Paruzel-Czachura et al. (2024). The rule-of-thumb proposed here thus is in harmony with children’s natural inclinations about animals’ value.

17 We thank an anonymous reviewer for pressing us on this point.

18 Our argument of course does not rule out the possibility of parents teaching their children further ways to reduce their environmental impact, such as avoiding flying.

19 See Baatz (2014, 2016) for a fair share argument regarding greenhouse gas emissions. He conceptualizes a fair share as the amount of goods one needs to live a decent life. Regardless of the view of a ‘fair share’ one endorses, one will likely agree that a typical affluent Westerner is using far more than their fair share.

20 The complementary nature of individual and structural aspects of climate change is well explained in Brownstein et al. (2022).

21 Note, however, that there may be morally significant differences between parents living in the very same place. For example, it may be harder for a financially struggling single mother to spend her limited funds buying plant-based products, whereas it may be easier for a family where one family member is at home full time and has time to prepare home-cooked meals for their children. We thank an anonymous reviewer for raising this issue.
